# The Mental Health Needs of Out-of-School Adolescents and Young Adults: An Intervention Conducted in Employment Training Programs, Baltimore, Maryland, 2007-2008

**Published:** 2012-03-08

**Authors:** S. Darius Tandon, Pallab K. Maulik, Margaret Gifford Tucker, Freya Sonenstein

**Affiliations:** Department of Pediatrics, Johns Hopkins University School of Medicine; The George Institute for Global Health, Hyderabad, India; Johns Hopkins University Bloomberg School of Public Health, Baltimore, Maryland; Johns Hopkins University Bloomberg School of Public Health, Baltimore, Maryland

## Abstract

**Introduction:**

Despite the large number of adolescents and young adults in employment training programs, a population that has poorer health and greater health risk than similarly aged in-school peers, we are unaware of any health interventions that have been evaluated in this setting. The primary objective of our study was to evaluate changes in depressive symptoms, coping strategies, and receipt of mental health services among low-income African American adolescents and young adults receiving a mental health intervention integrated into an employment training program.

**Methods:**

The intervention consisted of an on-site mental health clinician, a peer-led depression prevention intervention, and training sessions for employment training staff. A pretest-posttest design assessed depressive symptoms, coping strategies, and receipt of mental health services at baseline and 12-month follow-up. Complete baseline and follow-up data were available for 136 of 218 eligible participants. Most study participants were African American (98%); average age was 18.8 years.

**Results:**

The intervention had no effect on depressive symptoms or coping strategies. The percentage of participants who used mental health services at follow-up increased, but not significantly. Age was associated with use of active and support-seeking coping strategies, whereas use of mental health services before program enrollment was associated with use of mental health services at follow-up.

**Conclusion:**

Alternative intervention strategies may be needed to decrease the severity of depressive symptoms and increase use of coping strategies among adolescents and young adults in employment training programs. Future research evaluating such interventions should use quasi-experimental or experimental designs to provide evidence of intervention effect.

## Introduction

National community studies have found that 25% of adolescents and young adults aged 16 to 24 years will experience a depressive episode by age 24 — the highest incidence of any age group (1). Depression during adolescence and young adulthood has been associated with an increased likelihood of substance abuse, interpersonal problems, delinquency, academic and workplace difficulties, and suicide attempts (2-4). Moreover, people experiencing a depressive episode during adolescence or young adulthood are likely to experience another episode in later adulthood (5-7).

Sixty-five percent of African Americans aged 16 to 24 who have dropped out of school are unemployed (8). Many of these adolescents and young adults, in search of education opportunities and job training, are engaged in employment training programs. Health risks, especially mental health problems, are significant barriers to engaging in employment training programs (9). The Government Accountability Office found that lack of mental health services was among the most commonly identified gaps in the core services of employment training programs (10). Despite this gap, we are unaware of studies that examine interventions to improve access to mental health services and mental health status among adolescents and young adults in employment training programs.

The primary objective of our study was to evaluate changes in depressive symptoms, coping strategies, and receipt of mental health services among low-income African American adolescents and young adults receiving a mental health intervention integrated into an employment training program. The secondary objective was to examine individual and environmental variables associated with changes in study outcomes.

## Methods

### Setting

In 2000, the US Department of Labor awarded Youth Opportunity (YO) grants to 36 high-poverty areas (11) characterized by high rates of school dropout and unemployment. YO centers provide social and educational services to adolescents and young adults not currently in school or the workforce, including general educational development (GED) classes, resume building, and job placement. During 2005 and 2006, we collaborated with the Eastside YO Center, 1 of 2 YO programs in Baltimore, Maryland, to develop a mental health intervention. The collaboration included the Eastside YO Center's community advisory board and staff and a group of YO alumni. This study was conducted during 2007 and 2008.

### Study design

We used a pretest-posttest design to assess 3 outcomes: depressive symptoms, coping strategies, and receipt of mental health services. Study participants completed a baseline audio computer-assisted self-interview (ACASI) and a follow-up ACASI 12 months later.

### Study recruitment and sample

Between May 2007 and February 2008, 390 adolescents and young adults who were not in foster care enrolled at the Eastside YO Center. Of the 390 newly enrolling YO members, 323 (83%) completed a baseline ACASI designed to assess depressive symptoms, the use of coping strategies, and the use of mental health services and address other mental health and health topics. An ACASI allows a person to sit at a computer, hear questions on headphones while they appear on a screen, and use simple keyboard commands to respond. We used ACASIs to minimize concerns about participants' reading comprehension. In addition, ACASIs more accurately assess such stigmatizing behaviors as drug use and violence perpetration than paper-and-pencil self-administered surveys (12).

A research assistant obtained informed consent for participants aged 18 or older and youth assent and parental consent for younger participants. All procedures were approved by Johns Hopkins Bloomberg School of Public Health's Committee on Human Subjects Research.

We found no significant differences in race, sex, or age between participants completing baseline ACASIs and those enrolling in YO but not completing the ACASI. Of the 323 YO members completing the baseline ACASIs, 218 were eligible for the 12-month follow-up. The remaining 105 YO members were ineligible for the following reasons: incarceration at follow-up (n = 38), death (n = 1), moving out of state (n = 1), and never receiving services at YO (n = 65). Of the 218 eligible for follow-up, 136 (62%) completed 12-month ACASIs; these follow-up ACASIs also asked YO members about depressive symptoms, coping, and use of mental health services. We found no significant differences in race, sex, or age between the 136 YO members completing 12-month ACASIs and the 82 noncompleters.

### Mental health intervention

The mental health intervention consisted of 3 components.

**Referral to onsite mental health clinician**. After all baseline ACASIs were completed, a research assistant generated a short report on each participant's ACASI responses, and each participant was referred to the onsite clinical social worker. The short report was designed to provide the clinician with preliminary mental health information and help guide the first visit. The purpose of the referral was to familiarize the participant with the clinician; the referral process was hypothesized to facilitate subsequent receipt of mental health services. After assessment by the clinician, some participants were referred to an offsite mental health provider for additional services, including medication management.

**Depression prevention curriculum (SOS Club).** YO members who had a score of 10 to 26 on the Center for Epidemiologic Studies Depression Scale (CES-D) (13,14) were eligible to participate in the SOS Club, a 9-week intervention aimed at preventing the worsening of depressive symptoms. The curriculum was adapted from Structured Psychotherapy for Adolescents Responding to Chronic Stress, a skills-based curriculum designed to assist adolescents who have high levels of stress by promoting positive cognitive appraisals of stressful situations and enhancing coping skills (15,16). Each hour-long weekly session was delivered by trained peer leaders, who were YO graduates, and attended by a licensed clinician prepared to respond to potential safety issues that peer leaders were not trained to handle.

**Training for YO staff**. We provided 2-day training sessions to all YO staff to help them better understand the mental health issues of YO members, understand the role mental health plays in healthy adolescent development, engage in conversations about mental health with YO members, understand the range of mental health services available to YO members, and encourage YO members to obtain mental health services when appropriate. YO staff are employment and training specialists but have no formal education in mental health. The training sessions were led by several mental health researchers and practitioners. We provided these sessions to all YO staff and expected this component to have an effect on all YO members.

### Measures

**Depressive symptoms.** We used the CES-D (13) — a 20-item self-report instrument widely used in depression research with adolescents and young adults (17) — to measure the severity of depressive symptoms at baseline and follow-up as part of the ACASI. Each item is scored on a scale of 0 to 3; 4 items are reverse scored. The total score ranges from 0 to 60; higher scores indicate greater severity of depressive symptoms. We considered a CES-D score of 16 or more to indicate clinically meaningful depressive symptoms.

**Coping strategies.** The Children's Coping Strategies Checklist — Revision 1 (18) assessed 3 domains of engaged coping strategies at baseline and follow-up as part of the ACASI: active coping (eg, trying to figure out why things like this happen), support seeking (eg, telling people how you feel about a problem), and distraction (eg, listening to music). For each question, respondents were asked to indicate how often in the previous month they engaged in the coping strategy to solve their problems or make themselves feel better. Each item was scored from 1 ("never") to 4 ("most of the time"); scores were calculated by summing responses within each coping strategy. Active coping scores ranged from 0 to 92; distraction, 0 to 36; and support seeking, 0 to 36; higher scores indicate more engaged, or adaptive, coping.

**Receipt of mental health services**. We used the Service Assessment for Children and Adolescents (19) to assess receipt of mental health services in 3 domains at baseline and follow-up as part of the ACASI: inpatient, outpatient, and school-based services. Respondents indicated whether they had received mental health services from settings in each domain in the previous 12 months, excluding the initial visit with the onsite mental health clinician. Responses to these questions were yes and no.

**Individual and social environmental variables.** For all multivariate models, we examined age, sex, education level, baseline stress, and baseline social support as predictors of our dependent variables. Baseline stress was measured using the Stressful Life Events Scale (20); baseline social support was assessed using the Social Support for Adolescents Scale (21).

### Analysis

We summarized the sociodemographic characteristics of the sample. We calculated the mean CES-D score and mean coping strategies score for the sample at baseline and follow-up and conducted paired *t* tests to assess changes. We were especially interested in tracking the participants who had a CES-D score of 16 or more at baseline (ie, had clinically meaningful depressive symptoms upon enrollment). We used χ^2^ tests to compare the percentage of participants at baseline and follow-up with a mean CES-D score of 16 or more who reported receiving mental health services in the previous 12 months. Multivariate linear regression assessed individual and social environmental variables associated with changes in depressive symptoms and coping strategies between baseline and follow-up, and multivariate logistic regression assessed variables associated with receipt of mental health services at follow-up. We controlled for the baseline score for each outcome variable when we modeled that variable. We used splines for some independent variables based on the distribution of LOWESS (locally weighted scatterplot smoothing) plots of the independent and dependent variables. We checked collinearity for all models and chose the most parsimonious model based on Akaike information criteria. We used a 2-tailed α of .05 to define statistical significance. We analyzed data using STATA version 10 (StataCorp LP, College Station, Texas).

## Results

Nearly all (98%) study participants were African American; 45% were male; the average age was 18.8 (Table 1). Only 14% had a high school diploma or GED; most (71%) attended but did not complete high school. The mean (standard deviation [SD]) CES-D score at baseline was 15.0 (10.1). The mean (SD) level of baseline stress was 10.2 (5.5), and the mean (SD) level of baseline social support was 32.9 (5.7).

The mean CES-D score of 15.0 at baseline increased to 16.5 at 12-month follow-up (Table 2). Fifty-one participants had a CES-D score of 16 or more at baseline; of these, 22 (43%) had a score lower than 16 at follow-up. We found no significant changes in the use of coping strategies. At follow-up, 13 of 42 participants who had a CES-D score of 16 or more reported receiving mental health services in the previous 12 months, compared with 12 of 51 (24%) at baseline, although the difference was not significant.

A 1-point increase in the baseline stress score did not change the CES-D score significantly (Table 3). Age was a significant predictor of changes in active coping, when adjusted for other factors. This effect varied by age, however. Until age 21, the active coping score decreased by 0.81 (standard error [SE], 0.40; *P* = .047) for every year increase in age, but after age 21, the active coping score increased by 4.17 (SE, 1.76; *P* = .02) for every year increase in age. After age 18, the support-seeking coping score decreased by 0.92 (SE, 0.26; *P* = .001) for every year increase; the support-seeking coping score decreased by 0.11 (SE, 0.05, *P* = .03) for participants who had a baseline social support score higher than 25, after adjusting for other factors. Participants who received mental health services in the 12 months before baseline were almost 7 times as likely to receive services between baseline and follow-up.

## Discussion

Our intervention did not have an overall effect on reducing the severity of depressive symptoms or increasing the use of coping strategies. Among the 51 participants who entered the study with clinically meaningful depressive symptoms, 22 did not have such symptoms at follow-up. A decrease in symptomatology reduces the likelihood of substance abuse, interpersonal problems, and other behavioral concerns (22).

At lower levels of stress at baseline, the number of depressive symptoms decreased as stress increased. At a certain point, however, the number of depressive symptoms increased as stress increased. This finding is consistent with the "challenge" model of resilience (23), which proposes that the association between risk factors (eg, stressful events) and a health or well-being outcome (eg, depressive symptoms) is curvilinear: exposure to both low and high levels of a risk factor are more strongly associated with negative outcomes, but moderate levels of stress are associated with less negative outcomes. According to the challenge model, participants who had moderate levels of stress learned how to overcome these risks but were not exposed to so much stress that it was impossible to overcome its effects.

The use of active coping decreased for every year increase in age until 21 and then increased among participants aged 22 to 24. This pattern of decreasing active coping among younger members contradicts some research that identified increases in active coping during adolescence (24). Our study participants may have been exposed to more pervasive and acute stress than other adolescents, or they may have felt more frustrated or hopeless about the efficacy of active coping strategies in helping them to manage stressful life situations. These young people may have turned to other types of coping — eg, support seeking, distraction — if active coping strategies were not perceived as effective, although our data do not show whether our participants actually did this. Research shows that adolescents tend to use less active coping strategies when they perceive stressful situations to be particularly threatening or uncontrollable (25); our participants faced these kinds of situations.

Participants aged 18 or younger showed increases and older participants reported decreases in support-seeking coping as they got older. People living in stressful urban environments may seek out multiple sources of support as they grow older and are faced with more life stressors — including, in this population, disengagement from school and the workplace. However, as people mature, they may recognize the limitations of the people on whom they rely for social support and less frequently use support-seeking strategies. African American adolescents tend to rely on the same people (eg, extended family networks) to provide emotional, tangible, and informational support (26).

Some research suggests that adolescents and young adults use more support-seeking coping in school and peer contexts (27). The participants in our study did not attend school, so they lacked the school-based peer network that would have encouraged support seeking. The limited peer networks that exist for out-of-school youth validate the importance of peer-based approaches such as our depression-prevention intervention; peer-based approaches may be effective in encouraging older adolescents and young adults to actively seek and use various types of social support. Our finding that active and support-seeking coping strategies were used less frequently as participants aged also signals the importance of adapting intervention components for different age groups. For example, young adults may need encouragement and instruction to realize that just because past attempts to use active or support-seeking strategies did not work does not mean that future attempts will not work.

Our study had several limitations. We had a small sample, and depression can be cyclical, so findings on changes from baseline to follow-up should be interpreted cautiously. We did not use an experimental design to assess intervention effect; we believed that it was premature to use such a design because of the small amount of research available on the integration and evaluation of mental health services in employment training settings. The lack of a comparison group limits the interpretation of findings on the effect of the intervention on mental health outcomes. Our study was implemented at a single employment training program. Future studies could be implemented at multiple programs, thereby increasing the number of study participants and allowing greater generalizability of study results.

Study investigators and community partners learned several lessons, which have shaped the development and implementation of a more comprehensive mental health intervention currently being assessed through a quasi-experimental design. First, thorough training is needed to ensure that paraprofessionals — in our case, peer leaders — feel adequately prepared to deliver the intervention content. In our new intervention (28), peer leaders were observed by a research team member and found to have done an excellent job in both fully covering intervention content and delivering the content as it was presented in the instructor manual. Second, mental health services need to be integrated into regular employment training activities so participants are "touched" by mental health activities daily and health activities are not viewed as "special" services for a certain group of people but ongoing and accepted activities for all participants. Third, because we could not show overall intervention effects on depressive symptoms or coping strategies in this study, we developed a more targeted approach to providing mental health services in our new intervention. We now use baseline depressive symptom scores to triage participants according to 3 levels of depressive symptoms (low, moderate, and high). Clinicians work more with participants who have more depressive symptoms.

This the first study to our knowledge to examine the effects of an intervention aimed at improving the mental health of adolescents and young adults in employment training programs — a population shown to exhibit worse health and greater health risk than similarly aged in-school peers (27). This study suggests that alternative strategies — specially modeled for young people or targeted toward specific mental health concerns — may be needed to decrease the severity of depressive symptoms and increase use of coping strategies among adolescents and young adults in employment training programs. Future evaluations of such interventions should use more rigorous quasi-experimental or experimental designs to provide clearer evidence of intervention effect.

## Figures and Tables

**Table 1.** Characteristics of Participants (N = 136) at Baseline in a Study on the Mental Health Needs of Out-of-School Adolescents and Young Adults in an Employment Training Program, Baltimore, Maryland, 2007-2008

**Characteristic**	Value
**African American, n (%)**	134 (98.3)
**Sex,^a^ n (%)**	
Male	61 (44.9)
Female	74 (54.4)
**Age, mean (SD), y**	18.8 (2)
**Age range, y**	16-24
**Education, n (%)**	
Did not complete 9th grade	21 (15.4)
Completed 9th grade or more but did not obtain GED or high school diploma	96 (70.6)
Obtained GED or high school diploma	19 (14.0)
**CES-D^b^ score, mean (SD)**	15.0 (10.1)

Abbreviations: GED, general educational development; SD, standard deviation.

a One participant identified as transgendered.

b Center for Epidemiologic Studies Depression Scale (13). The score ranges from 0-60; higher scores indicate greater severity of depressive symptoms.

**Table 2.** Mental Health Characteristics at Baseline and Follow-Up, Participants (N = 136) in a Study on the Mental Health Needs of Out-of-School Adolescents and Young Adults in an Employment Training Program, Baltimore, Maryland, 2007-2008

**Characteristic**	Baseline	12-Month Follow-Up	*P* Value
**CES-D score,^a^ mean (SD)**	15.0 (10.1)	16.5 (11.2)	.19
**Participants who had CES-D score ≥16,^b^ n (%)**	51 (37.5)	42 (30.9)	NC
**Coping strategies score,^c^ mean (SD)**			
Active	31.1 (6.8)	30.5 (7.6)	.41
Distraction	10.3 (2.2)	9.9 (2.1)	.40
Support seeking	10.0 (3.2)	10.2 (2.9)	.52
**Receipt of mental health services among those who have CES-D score ≥16,^d^ n (%)**	12/51 (24)	13/42 (31)	.26

Abbreviations: CES-D, Center for Epidemiologic Studies Depression Scale (13); SD, standard deviation; NC, not calculated.

a Determined by CES-D (13). Scores range from 0-60; higher scores indicate greater severity of depressive symptoms. Change between baseline and follow-up was determined by paired *t* tests.

b A CES-D score of ≥16 was considered to indicate clinically meaningful depressive symptoms.

c Determined by The Children's Coping Strategies Checklist — Revision 1 (18). Active coping scores ranged from 0-92; distraction, 0-36; support seeking, 0-36; higher scores indicate more engaged, or adaptive, coping.

d Determined by the Service Assessment for Children and Adolescents (19). Possible responses were yes and no.

**Table 3.** Multivariate Analysis for Study on the Mental Health Needs of Out-of-School Adolescents and Young Adults (N = 136) in an Employment Training Program, Baltimore, Maryland, 2007-2008.

Linear Models		

**Model/Variable**	β (SE)	*P* Value
**Model 1: Depressive symptoms at 12 months**		
Baseline stress score^a^ ≤8	−0.82 (0.54)	.13
Baseline stress score^a^ >8	0.24 (0.26)	.37
**Model 2: Active coping score at 12 months**		
Female	1.27 (1.21)	.30
Age ≤21 y	−0.81 (0.40)	.047
Age >21 y	4.17 (1.76)	.02
**Model 3: Support-seeking coping score at 12 months**		
Female	−0.09 (0.48)	.85
Age ≤18 y	0.43 (0.39)	.27
Age >18 y	−0.92 (0.26)	.001
Baseline social support^b^ ≤25	0.53 (0.28)	.06
Baseline social support^b^ >25	−0.11 (0.05)	.03
**Model 4: Distraction coping score at 12 months**		
Female	0.53 (0.33)	.11

**Logistic Model**		

**Model**	**Odds Ratio (95% CI)**	***P* value**

**Model 5: Receipt of mental health services between baseline and follow-up, reported at 12 months**		
Any service use in previous 12 months reported at baseline	6.78 (2.16-21.33)	.001
Any insurance reported at baseline	0.87 (0.34-2.20)	.77
Total baseline social support	1.05 (0.96-1.14)	.27

Abbreviations: SE, standard error; CI, confidence interval.

a Determined by the Stressful Life Events Scale (20). Scale ranges from 0-29; the higher the score, the more stress in the past 12 months.

b Determined by the Social Support for Adolescents Scale (21). Scale ranges from 18-54; the higher the score, the more social support.
